# Relationships between Obesity, Exercise Preferences, and Related Social Cognitive Theory Variables among Breast Cancer Survivors

**DOI:** 10.3390/nu15051286

**Published:** 2023-03-04

**Authors:** Nashira I. Brown, Dorothy W. Pekmezi, Robert A. Oster, Kerry S. Courneya, Edward McAuley, Diane K. Ehlers, Siobhan M. Phillips, Philip Anton, Laura Q. Rogers

**Affiliations:** 1 Department of Health Behavior, University of Alabama at Birmingham (UAB), Birmingham, AL 35294, USA; 2 O’Neal Comprehensive Cancer Center at UAB, Birmingham, AL 35233, USA; 3 Department of Medicine, Division of Preventive Medicine, Birmingham, AL 35294, USA; 4 Faculty of Kinesiology, Sport, and Recreation, University of Alberta, Edmonton, AB T6G 2H9, Canada; 5 Department of Kinesiology and Community Health, University of Illinois at Urbana-Champaign, Urbana, IL 61801, USA; 6 The Cancer Center at Illinois, Urbana, IL 61801, USA; 7 Department of Quantitative Health Sciences, Mayo Clinic, Phoenix, AZ 85054, USA; 8 Department of Preventive Medicine, Northwestern University Feinberg School of Medicine, Chicago, IL 60611, USA; 9 School of Human Sciences, Southern Illinois University Carbondale, Carbondale, IL 62901, USA

**Keywords:** physical activity, obesity, cancer survivors, preferences

## Abstract

Breast cancer survivors with obesity have an increased risk of cancer recurrence, second malignancy, and comorbidities. Though physical activity (PA) interventions are needed, investigation of the relationships between obesity and factors influencing PA program aspects among cancer survivors remain understudied. Thus, we conducted a cross-sectional study examining associations amongst baseline body mass index (BMI), PA program preferences, PA, cardiorespiratory fitness, and related social cognitive theory variables (self-efficacy, exercise barriers interference, social support, positive and negative outcome expectations) from a randomized controlled PA trial with 320 post-treatment breast cancer survivors. BMI was significantly correlated with exercise barriers interference (*r* = 0.131, *p* = 0.019). Higher BMI was significantly associated with preference to exercise at a facility (*p* = 0.038), lower cardiorespiratory fitness (*p* < 0.001), lower walking self-efficacy (*p* < 0.001), and higher negative outcome expectations (*p* = 0.024), independent of covariates (comorbidity score, Western Ontario and McMaster Universities osteoarthritis index score, income, race, education). Those with class I/II obesity reported a higher negative outcome expectations score compared with class III. Location, walking self-efficacy, barriers, negative outcome expectations, and fitness should be considered when designing future PA programs among breast cancer survivors with obesity.

## 1. Introduction

The prevalence of breast cancer survivors continues to grow as diagnosis, treatment, and control advance. However, quality of life after diagnosis among this population is affected by modifiable lifestyle behaviors, including physical activity (PA) engagement [1]. American Cancer Society Guidelines for PA recommend at least 150 min of exercise per week and 2 days of strength training per week for cancer survivors to help reduce risk of cancer recurrence, second malignancy, and comorbidities (e.g., obesity) [2]. Notably, a minority (13.9%) of breast cancer survivors meet PA guidelines and only 35% maintain a healthy BMI [3]. Moreover, adult cancer survivors experience a more rapid rise in obesity [4], which has been associated with low cardiorespiratory fitness, a strong predictor of all-cause and cardiovascular mortality, following cancer treatment(s) [5], and thus are in need of effective lifestyle intervention [4]. 

Best practices for assisting this population in adopting a physically active lifestyle include using evidence-based theories [6] and program preferences [7] for PA program design. The social cognitive theory (SCT) is one of the most widely applied health behavior theories in PA research [8,9]. This framework posits that behavior is a dynamic interaction of a triad of factors (i.e., personal cognitive, the physical and social environment (socioenvironmental), and behavioral). SCT constructs that are commonly targeted in PA interventions are self-efficacy, exercise barriers interference, outcome expectations, and social support. Self-efficacy, or the confidence in one’s ability to take action and overcome obstacles and situations to reach a goal, is deemed as the significant primary personal factor that mediates behavior change [8], especially PA behaviors in women with breast cancer [10]. This construct has been associated with body mass index (BMI) in cancer survivors [11,12], along with other constructs, such as social support and exercise barriers interference [12]. 

In addition to SCT constructs, research focusing on what individuals prefer could be vital to optimizing participant engagement and acceptability when designing PA programs [12]. Although multiple studies have reported PA preferences for breast cancer survivors [13], very few have examined how preferences may vary by level of obesity [12,14]. Further, the few studies in populations without a history of cancer have shown that PA preferences (i.e., intervention delivery, supervision, and scheduling) differed among those with and without obesity [15,16]. Given the paucity of research on the relationship between obesity and potential differences in preferences among breast cancer survivors with cancer, further examination is warranted to develop effective PA programs. 

Thus, to inform future PA promotion programs for breast cancer survivor populations with obesity, we performed a secondary analysis of baseline data from a randomized PA intervention trial [17]. Our study purpose was to examine the associations between BMI and factors influencing program content and delivery preferences (source, mode, structure, location, furthest distance willing to travel, furthest distance willing to travel if someone else paid for gas, price willing to pay for exercise program, program type, supervision, alone or with group), current PA (accelerometer), cardiorespiratory fitness, and related SCT constructs (i.e., self-efficacy (barriers and task), exercise barriers interference, social support, outcome expectations (negative outcome expectations and positive outcome expectations)) among the enrolled breast cancer survivors (*n* = 320). We hypothesized that BMI would be significantly associated with program preferences, PA, cardiorespiratory fitness, and related SCT constructs. 

## 2. Materials and Methods

### 2.1. Study Design

This cross-sectional study examined relationships amongst BMI, PA program preferences, PA, cardiorespiratory fitness, and related SCT variables. Data for these secondary analyses were taken from the baseline survey for a randomized controlled PA behavior change trial with 320 post-treatment breast cancer survivors. Participants enrolled in the primary trial (Better exercise adherence after treatment for cancer (BEAT Cancer); *n* = 222) [18] were combined with participants enrolled to a trial supplement (accelerometer calibration sub-study entitled “Comparing doubly-labeled water to accelerometer to assess PA measurement error during and after a physical activity behavior change intervention” (COMPARE); *n* = 98).

### 2.2. Study Sample 

Three hundred and twenty post-primary treatment breast cancer survivors were recruited through newspaper advertisements, cancer support groups, flyers posted in relevant locations (e.g., hospitals, physician offices, cancer centers/clinics), and areas frequented by women (e.g., retail stores, beauty salons). Eligible women met the following criteria: English speaking, between the ages of 18 and 70 years of age with a history of ductal carcinoma in situ (DCIS) or Stage I, II, or IIIA breast cancer and post-primary chemotherapy or radiation therapy, medically cleared for participation by their physician and underactive (participating in no more than 60 min of moderate intensity PA or no more than 30 min of vigorous intensity activity per week, on average, over the past 6 months).

## 3. Measures

### 3.1. Demographics and BMI

Self-reported participant demographics included age, race, ethnicity, years of education, annual household income, employment status, marital status, cancer stage at diagnosis, history of chemotherapy, history of radiation therapy, hormonal therapy type, functional comorbidity index score [19], and the Western Ontario and McMaster Universities osteoarthritis index (WOMAC) [20]. The functional comorbidity index score, or the number of comorbidities, was assessed by totaling the number of “yes” responses to 18 diagnoses with possible scores of 0–18, 0 indicating no comorbidities and 18 indicating the highest number of comorbidities [19]. The WOMAC, 24-item scale, assessed lower extremity joint pain (5 items), stiffness (2 items), and physical dysfunction (17 items) [20]. Scores from the subscales were summed with possible score ranging from 0 to 68, with higher scores indicating greater pain, stiffness, and physical dysfunction [20]. Weight and height were measured in person by trained research staff using a calibrated scale and stadiometer. Brand and model were study site specific (University of Alabama at Birmingham (Detecto Model 439); Southern Illinois University(Continental Health-O-Meter #400 DML medical scale); University of Illinois (Seca 763 Digital Column Scale)) [21]. BMI was calculated using the measured weight and height (weight (kg)/height (m^2^)) [21].

### 3.2. Exercise Program Preferences

Exercise program preferences were assessed using a 15-item multiple choice self-administered survey that has been used in prior studies among breast cancer survivors [12,14,22,23]. The counseling preference items included queries regarding counseling source (i.e., cancer exercise physiologist, personal trainer, medical doctor, nurse, health club exercise specialist, cancer patient/survivor), mode of delivery (i.e., face-to-face, phone, video, written material, internet, audiotape, interactive workbook), and company (i.e., individual or with a group). Exercise training preference items focused on location (i.e., at home, outdoors, at work, health club, cancer exercise center), exercise type (i.e., walking, water, bike, jogging, resistance, yoga, Pilates), and supervision (i.e., supervised, or unsupervised). Programming preference items inquired about program type (i.e., aerobic, strength, or both), structure (i.e., flexible vs. scheduled), maximum price willing to pay for an exercise program (i.e., $0, $1–10/month, $11–20/month, $21–30/month, $31–40/month, $40+/month), farthest distance willing to travel to an exercise program (0 miles, 1–15 miles, 16–30 miles, 31–45 miles, 46–60 miles, 60+ miles), and the farthest distance willing to travel to an exercise program if cost of gas was covered (0 miles, 1–15 miles, 16–30 miles, 31–45 miles, 46–60 miles, 60+ miles). 

### 3.3. PA

Weekly minutes of moderate-plus-vigorous intensity PA were assessed with ActiGraph accelerometer (model: GT3X, Pensacola, FL, USA). Participants were instructed (orally and written) to wear the device for at least 10 waking hours for seven (primary trial) or 10 (COMPARE) consecutive days [24]. The parameters used to validate the minimum wear time of 4 days was comprised of wear time ≥10 waking hours. The cut points that were used to establish moderate-to-vigorous activity intensity were: moderate (1952–5724 counts/min) and vigorous (5725+ counts/min) [21]. Minutes of vigorous intensity activity were doubled prior to adding minutes of moderate intensity activity and calculating weekly minutes of moderate-to-vigorous activity. 

### 3.4. Cardiorespiratory Fitness

Following the American College of Sports Medicine guidelines for testing [25], cardiorespiratory fitness (relative VO_2_ peak) was estimated with submaximal treadmill testing [26] in which speed and elevation were gradually increased until the participant achieved 85% of age-predicted maximal heart rate. Following the modified Naughton protocol, tests were begun at a slower speed and progressed at lower increments, as in past studies with individuals who are sedentary, older, fatigued, or have balance complications [21,25]. The oxygen cost of walking at the treadmill grade and speed achieved at 85% of predicted heart rate was estimated using published regression equations and is expressed in mL/kg/min [27]. 

### 3.5. PA Related SCT Variables

#### 3.5.1. Self-Efficacy

Both barriers and task self-efficacy were assessed. One’s confidence in his/her ability to act and overcome obstacles and situations to reach a goal was assessed using a reliable (α = 0.97–0.98) 9-item scale, Barriers Self-Efficacy, designed for breast cancer patients [28]. Walking task self-efficacy was assessed using a valid and reliable (*r* = 0.89 and α = 0.96) 6-item scale, Self-Efficacy for Walking, to measure confidence in walking at a moderate pace for six different intervals of time (i.e., 5, 10, 15, 20, 25, and 30 min) [29]. Both measures of self-efficacy asked participants to indicate their confidence (0–100%, at 10% intervals (i.e., not at all confident, 0–20%; slightly confident, 20–40%; moderately confident, 40–60%; very confident, 60–80%; extremely confident, 80–100)) [28,30]. Responses were averaged separately for barriers and task self-efficacy with a range of possible scores (0–100). 

#### 3.5.2. Exercise Barriers Interference

Perceived barriers (or barriers interference), or how often recognized obstacles (i.e., lack of time, fear of injury, fatigue, lack of energy, lack of company, cost of exercising, lack of enjoyment, lack of equipment, family responsibilities, inconvenient exercise schedule, lack of interest, lack of knowledgeable exercise staff, feeling nauseated, no facilities/space, not a priority, procrastination, pain/discomfort, not in routine, lack of self-discipline, lack of skills, weather) interfered with exercise, was assessed using a 21-item, 5-point Likert scale (1 = rare to 5 = very often) measure, Exercise Barriers Interference, that has demonstrated reliability (α = 0.92) among breast cancer survivors [31]. Responses were summed for a total exercise barriers interference score [31,32,33] with a range of possible scores (21–105). 

#### 3.5.3. Social Support

Social support, or the perception of encouragement to engage in PA, from other sources (i.e., friends and family) [31] was measured via a 4-item (friends, 2-items, family, 2-items), 5-point Likert scale (0 = none to 4 = very often), Social Support for Physical Activity, with an internal consistency of 0.80 [31]. Responses were summed for a total social support score with possible scores ranging from 0 to 16 [34,35]. 

#### 3.5.4. Outcome Expectations

Outcome expectations, or the anticipated positive and/or negative consequences of engaging in a behavior (e.g., exercise) was evaluated using a reliable (α = 0.79 and 0.70, respectively) 17-item (14 positive expectations and 3 negative expectations), 5-point Likert scale (1 = strongly disagree to 5 = strongly agree); responses were summed for positive and negative outcomes separately (i.e., higher score indicates greater perceived benefit (positive expectations) or greater perceived risk (negative expectations)) with possible scores ranging from 14 to 70 and 3 to 15, respectively [32]. 

#### 3.5.5. Statistical Analyses

Descriptive analyses were conducted to summarize participant characteristics. BMI was analyzed as a continuous variable and as a 3-level categorical outcome (i.e., non-obese (BMI ≤ 29.9 kg/m^2^), obese classes I/II (BMI = 30–39.9 kg/m^2^), and obese class III (BMI ≥ 40 kg/m^2^)). A 3-level BMI was created to facilitate identifying potentially important differences at a higher BMI that may be missed when analyzing with a continuous BMI.

The associations with continuous BMI were analyzed using Pearson correlation coefficients (continuous correlates) and independent groups t-test (continuous correlates). The associations with 3-level categorical BMI were analyzed using one-way analysis of variance (ANOVA), followed by the Tukey *post-hoc* test (continuous correlates), and also the chi-square test (categorical or dichotomous correlates). Continuous study variables were examined for normality of distribution using box plots, stem-and-leaf plots, normal probability plots, and the Kolmogorov–Smirnov test. Variables that were determined as being non-normally distributed were also examined using the non-parametric Kruskall–Wallis test. Since the parametric were similar to the non-parametric results, we report the parametric results for ease of interpretation, and previous studies have reported parametric results obtained from these or similar variables. Follow-up multiple linear or logistic regression were performed based on the type of dependent variable. 

No imputations were performed for missing data since the amount of missing data were very small (<1%); there were no missing data for most of the study variables. All analyses were performed using IBM SPSS Statistics software (SPSS) Version 28 (Armonk, NY, USA: IBM Corp), and *p* < 0.05 was deemed as statistically significant. 

Exercise program preferences were dichotomized due to the small stratum-specific sample sizes with an emphasis on preferences more likely to alter program design (e.g., facility vs. other). Hence, each preference was reviewed based on the number of participants preferring each option within a specific preference question and then collapsed based on preference with greater potential to alter a program design (e.g., facility vs. other options not requiring a facility). 

Accelerometer-measured PA was analyzed as dichotomous, categorical outcome (met PA recommendations: >150 min of moderate-to-vigorous PA vs. did not meet PA recommendations: <150 min of moderate-to-vigorous PA) based on the American Cancer Society guidelines for PA for cancer survivors and to simplify interpretation [2].

### 3.6. Covariates and Adjusted Analyses

The variables investigated as potential covariates included age, race, ethnicity, education, income, employment, marital status, cancer stage at diagnosis, months since diagnosis, history of chemotherapy, history of radiation, hormonal therapy, number of comorbidities, and WOMAC score. A variable was considered a covariate if it was statistically significantly associated with both BMI and one or more of the correlates of interest (PA preferences, PA, cardiorespiratory fitness, and SCT variables). The identified covariates were then used for adjusted multiple variable regression analyses performed as indicated, with linear regression analysis performed for continuous outcomes and logistic regression analysis performed for dichotomous outcomes. All regression coefficients were tested for statistical significance. The *R*^2^ value was examined as goodness of fit measure.

## 4. Results

### 4.1. Participants

The participant characteristics are summarized in Table 1. Overall, participants were post-treatment breast cancer survivors with over half (52.2%) having obesity (mean BMI of 31.1 ± 7.34 kg/m^2^). Most participants were white (83%), non-Hispanic (98.7%), employed (67.5%) with an annual household income greater than $50,000 (67.6%), and a history of chemotherapy (61.6%) or radiation (65.6%). At cancer diagnosis, most were stage 1 (39.1%).

### 4.2. BMI and Program Preferences

A statistically significant relationship between BMI and a dichotomized program preference was found for only one preference, regardless of whether BMI was analyzed as a continuous variable (*p* = 0.009) or as a 3-level outcome (*p* = 0.038). Participants who preferred to exercise at a facility had a higher BMI (versus lower) (32.8 ± 8.6 vs. 30.3 ± 6.6 respectively). No other program preferences were associated with the continuous or 3-level BMI outcomes.

### 4.3. BMI, Current PA, and Cardiorespiratory Fitness

There were no statistically significant differences or associations with levels of current PA (moderate-to-vigorous PA or meets recommendations) when BMI was analyzed as a 3-level categorical outcome (Table 2 and Table 3). Similarly, no statistically significant association between PA and continuous BMI was noted (Table 4). However, there was a significant inverse correlation between BMI and cardiorespiratory fitness (*r* = −0.414, *p* < 0.001) in which higher BMI was related to lower cardiorespiratory fitness (Table 4). Results from a one-way ANOVA yielded statistically significant differences in cardiorespiratory fitness between levels of BMI (Table 2). A Tukey *post-hoc* test indicated that there was a significant difference in cardiorespiratory fitness between all levels of BMI (all *p* < 0.001). Specifically, cardiorespiratory fitness was significantly lower among obese class III vs. non-obese and obese class I/II.

### 4.4. BMI and SCT Variables

Pearson correlation coefficients between continuous BMI and PA-related SCT variables are provided in Table 4. Higher BMI was significantly correlated with higher exercise barriers interference (*r* = 0.131, *p* = 0.019) and lower walking self-efficacy (*r* = −0.364, *p* < 0.001). No other SCT variables were significantly correlated with BMI.

The one-way ANOVA showed statistically significant differences in walking self-efficacy and negative outcome expectations as reflected in Table 2. A Tukey *post-hoc* test indicated that there was a significant difference in walking self-efficacy scores between all levels of BMI (all *p* < 0.001). Walking self-efficacy scores were significantly lower among obese class III vs. non-obese and obese class I/II. As for the negative outcome expectations, obese class I/II reported significantly higher negative outcome expectations than non-obese (*p* = 0.024). The remaining constructs did not show any statistically significant differences.

### 4.5. Adjusted Associations

Multiple variable linear regression analyses were performed to examine the independent relationships between BMI (dependent variable) and fitness, exercise barriers interference, walking self-efficacy, and identified covariates (comorbidity score, WOMAC, income, race, and education). The model was statistically significant (*F*(9, 306) = 15.875, *p* < 0.001, *R*^2^ = 0.318) indicating that walking self-efficacy, cardiorespiratory fitness, comorbidity score, and race are independently associated with BMI (*p* < 0.05). Table 5 provides the analyses results.

### 4.6. Logistic Regression Models

Because preferring a facility was associated with BMI and walking self-efficacy, a *post-hoc* binomial logistic regression was performed to determine the relationship between location preference (dependent variable) and BMI independent of walking self-efficacy. The adjusted logistic regression model including BMI, walking self-efficacy, and location was statistically significant, χ^2^(2) = 10.292, *p* = 0.006. For every 1-unit increase in BMI, there is a 1.04 greater odds of preferring to exercise at a facility independent of walking self-efficacy score with a 95% confidence interval of (1.001–1.073).

## 5. Discussion

In this study, we sought to examine the associations between BMI and factors influencing program content and delivery (preferences), current PA (accelerometer-measured), cardiorespiratory fitness, and related SCT constructs (i.e., self-efficacy (barriers and task), exercise barriers interference, social support, outcome expectations (negative outcome expectations and positive outcome expectations)) among breast cancer survivors. We found that BMI was significantly correlated with exercise barriers interference. Further, we found significant associations between higher BMI and preference to exercise at a facility, lower cardiorespiratory fitness, and lower walking self-efficacy, independent of covariates (comorbidity score, Western Ontario and McMaster Universities osteoarthritis index score, income, race, education). These findings suggest that BMI influences PA program preferences, multiple psychosocial factors, and fitness which could be useful for informing future PA interventions for this population.

Overall, our sample of breast cancer survivors preferred to exercise at home or had no preference, which is consistent with the existing literature among diverse cancer survivors [36]. A recent systematic review reported a similar finding that adults with obesity prefer to engage in exercise “close to home”, while Hussien et al. found that adults with severe obesity prefer exercising outdoors [37]. However, when assessing preference by BMI category, we found that as obesity increased (i.e., obese class I/II to class III), there was an increased preference for exercising at a facility. This is similar to findings from another study among rural breast cancer survivors with overweight and obesity [38]. Potential hypotheses are that survivors with obesity may appreciate the support/supervision of facility staff (e.g., personal trainers, health coaches, health club exercise specialist), facility-provided equipment that limits weight bearing, and social context (i.e., seeing others exercise). Further, breast cancer survivors were recruited to participate in the current study, which required in-person visits to a facility.

The current study did not find a statistically significant association between BMI and current PA, similar to a past study among adults with obesity and multiple sclerosis [39]. Our sample consisted of breast cancer survivors who self-reported that they were not currently active (i.e., engaging in no more than 60 min of moderate intensity PA or no more than 30 min of vigorous intensity activity per week), as noted earlier in the inclusion criteria, which may have impacted our ability to test this relationship. However, a majority of the sample met PA recommendations based on free-living PA measured by accelerometer. We did find that overall, breast cancer survivors with and without obesity had low cardiorespiratory fitness levels compared to healthy women without cancer [40]. However, those with higher BMI had significantly lower cardiorespiratory fitness as in past studies among adults with obesity and no history of cancer [41,42]. Engaging cancer survivors, especially those with obesity, in cardiorespiratory fitness enhancing activities (i.e., PA, weight loss) is critical [43].

We found a statistically significant relationship between BMI and several SCT constructs. Specifically, breast cancer survivors with higher BMI had significantly lower walking self-efficacy and higher exercise barriers interference scores. Past studies have found associations between BMI and exercise barriers interference [44], exercise self-efficacy [45], and family social support [46] among adults with obesity, no cancer history [46], and presence of chronic illness (i.e., multiple sclerosis) [39]. The lower levels of self-reported walking self-efficacy among cancer survivors with higher BMI in the current study could possibly be due in part to limited mobility caused by excess body weight and reduced fitness levels, which makes it more difficult to walk at a moderately fast pace without stopping. With regard to exercise barriers interference, participants with increased weight reported more barriers to PA which has been previously described [15]. Similar to walking self-efficacy, bearing more body weight increases the likelihood of barriers (i.e., lack of energy, lack of confidence, lack of enjoyment, lack of interest, pain/discomfort).

### 5.1. Clinical and Research Implications

The findings from the current study have important implications for clinicians, healthcare professionals, and researchers who prescribe exercise and develop PA interventions for breast cancer survivors, especially with obesity. When prescribing exercise/PA for this vulnerable population, clinicians need to individually assess and consider medical (i.e., BMI), preference (i.e., location), SCT (i.e., walking self-efficacy), and fitness-related (i.e., cardiorespiratory fitness) factors as this could influence participation and the likelihood of meeting PA recommendations.

Although breast cancer survivors with obesity were more likely to prefer to engage in exercise at a facility (versus at home or no preference), potential barriers (e.g., financial, lack of accessibility) may arise for those without facility access. Therefore, distance-delivered interventions (e.g., mHealth, telephone, virtual) could serve as alternative options for delivering such programs. As for walking self-efficacy, exercise/PA programs can target and improve this component through incorporating individual behavior change techniques (e.g., goal setting, self-monitoring, and implementing solutions) [47,48]. The low levels of fitness found in the current study are more indicative of a greater need for PA and a potential intervention target. Thus, this suggests that structured exercise progression focused on improving cardiorespiratory fitness may be needed for this group. Lastly, although our study does not assess strength training, future interventions could leverage a preference for facility-based exercise to increase this type of exercise. In turn, individuals with obesity may be better able to do strength training at first, with a progression to greater aerobic exercise as muscle strength improves.

Taken as a whole, future research efforts need to be aimed at gaining a nuanced understanding regarding specific preferences (e.g., Why is facility more preferred by survivors with obesity?) through a qualitative approach. Moreover, future research directions should include assessing whether tailoring interventions and programs to individual health-related factors and preferences increases active living among this population.

### 5.2. Strengths and Limitations

The findings of the current study provide insight on a population that might need tailored or targeted PA programing. Specifically, our data related to location preference and health-related factors (i.e., walking self-efficacy and cardiorespiratory fitness) that can affect participation in programs can be useful for future intervention design. However, there are limitations. First, the entire sample consisted of post-treatment cancer survivors, mainly educated, affluent Caucasian women. Hence, findings may not be generalizable to the larger population of cancer survivors (e.g., those who are not White, have not undergone treatment, or are currently receiving treatment, less educated, less affluent, or male). Furthermore, although we used accelerometers to objectively measure minutes of PA, there are limitations (i.e., estimation of minutes of sedentary behaviors and PA). For example, it is unclear whether the device (waist-worn) accurately distinguishes the difference between sitting and standing idle, and upper body movement [49,50]. Moreover, accelerometers assess free-living PA rather than volitional, which results in a higher prevalence of meeting PA recommendations which are often based on leisure-time exercise alone. Lastly, our sample consisted of fewer participants with class III obesity (than with class I/II), which warrants future research with more representation from this group.

## 6. Conclusions

Cancer survivors are living longer due to advances in the diagnosis and control of cancer. Healthy lifestyle habits (physical activity, weight control) can enhance the quality of these years by reducing risk of cancer recurrence and other chronic diseases. Effective lifestyle programs are needed and should take into consideration the physical activity preferences and potential influences specific to this population, particularly among those with obesity who are most in need. Location, walking self-efficacy, and fitness are factors that should be considered for future PA programs among breast cancer survivors who have obesity. Tailoring for such individuals should involve a theoretically driven program which targets walking self-efficacy and involves activities appropriate for various levels of cardiorespiratory fitness.

## Figures and Tables

**Table 1:** Overall sample characterization (*n* = 320).

Characteristic	Overall (*n* = 320)
Age (years) (mean ± SD (range))	54.8 ± 8.3 (21–70)
Race *n* (%)	
Caucasian	256 (80)
African American	48 (15)
Other	16(5)
Ethnicity *n* (%)	
Non-Hispanic	315 (99)
Education (years) (mean ± SD (range))	15.5 ± 2.5 (9–21)
Income *n* (%)	
>$50 K	214 (68)
Employed *n* (%)	216 (68)
Marital Status *n* (%)	
Married or living with significant other	221 (69)
Widowed/Divorced/Single	99 (31)
BMI (mean ± SD (range))	31.1 ± 7.3 (18–58)
Non-obese *n* (%)	152 (48)
Obese Classes I/II *n* (%)	129 (40)
Obese Class III *n* (%)	39 (12)
Weekly minutes of Moderate-to-Vigorous PA (mean ± SD (range)) 173.6 ± 105.5 (30–1016)	
Cardiorespiratory Fitness (mL/kg/min) (mean ± SD (range)) 20.7 ± 5.1 (6–39)	
Cancer Stage at Diagnosis *n* (%)	
DCIS	40 (13)
I	125 (39.1)
II	121 (38)
III	33 (10.3)
Months since diagnosis (mean ± SD (range))	53.4 ± 54.2 (2–276)
History of chemotherapy *n* (%)	197 (62)
History of radiation *n* (%)	210 (66)
Hormonal Therapy (type) *n* (%)	
Estrogen receptor modulator	75 (23)
Aromatase inhibitor	85 (27)
None	160 (50)
Number of comorbidities (mean ± SD (range))	2.2 ± 1.8 (0–18)
WOMAC score (mean ± SD (range))	16.7 ± 15.0 (0–68)

Note: WOMAC score indicates lower extremity joint pain, stiffness, and dysfunction. Abbreviations: BMI, body mass index; DCIS, ductal carcinoma in situ; PA, physical activity; SD, standard deviation; WOMAC, Western Ontario and McMaster Universities osteoarthritis index.

**Table 2:** Differences in moderate-to-vigorous intensity PA, cardiorespiratory fitness, and SCT variables between the three levels of BMI.

	Group	Mean	SD	*F*(2, 317)	*p*-Value
Moderate-to-Vigorous Intensity PA (Weekly minutes)	Non-Obese	172.7	110.4	0.356	0.701
	Obese Class I/II	178.3	97.5		
	Obese Class III	162	112.4		
Cardiorespiratory Fitness (VO_2_peak; mL/kg/minute)	Non-Obese	22.4	4.5	35.175	<0.001
	Obese Class I/II	20.2	4.9		
	Obese Class III	15.7	3.8		
Exercise Barriers Interference (Score, possible range 0 to 100)	Non-obese	56.8	11.8	1.869	0.156
	Obese Class I/II	59.4	13.4		
	Obese Class III	59.9	13.3		
Walking Self-Efficacy (Score, possible range 0 to 100)	Non-obese	77.6	22.7	22.587	<0.001
	Obese Class I/II	65.9	22.2		
	Obese Class III	49.8	30.7		
Barriers Self-Efficacy (Score, possible range 21 to 105)	Non-obese	38.6	21.4	0.042	0.959
	Obese Class I/II	39.3	20.3		
	Obese Class III	39.3	25.7		
Positive Outcome Expectations (Score, possible range 14 to 70)	Non-obese	58.1	6.2	0.129	0.879
	Obese Class I/II	57.8	6.2		
	Obese Class III	57.7	7.7		
Negative Outcome Expectations (Score, possible range 3 to 15)	Non-obese	7.6	2.6	3.756	0.024
	Obese Class I/II	8.4	2.8		
	Obese Class III	7.7	2.7		
Total Social Support (Score, possible range 0 to 16)	Non-obese	4.5	4.3	0.845	0.430
	Obese Class I/II	4.0	3.9		
	Obese Class III	4.9	3.9		

Abbreviations: PA, physical activity; SD, standard deviation.

**Table 3:** Crosstabulations and chi-square tests for three-level BMI and meeting PA recommendations.

Moderate-to-Vigorous Intensity PA						
Group	Total (*n*)	Met Recommendations *n* (Row%)	Did Not Meet Recommendations *n* (Row%)	*χ* ^2^	*df*	*p*-Value
Non-obese	146	70 (48)	76 (52)	2.446	2	0.294
Obese Class I/II	121	68 (56)	53 (44)			
Obese Class III	38	17 (45)	21 (55)			

Note: Met PA recommendations: >150 min of moderate-to-vigorous PA vs. did not meet PA recommendations: <150 min of moderate-to-vigorous PA.

**Table 4:** Pearson correlation matrix for BMI, moderate-to-vigorous PA, cardiorespiratory fitness, and SCT variables.

	BMI	MVPA	Cardiorespiratory Fitness	Exercise Barriers Interference	Walking Self-Efficacy	Barriers Self-Efficacy	Positive Outcome Expectations	Negative Outcome Expectations	Total Social Support
BMI									
MVPA	−0.043								
Cardiorespiratory Fitness	−0.431 **	0.040							
Exercise Barriers	0.131 *	−0.117 *	−0.137 *						
Walking Self-Efficacy	−0.391 **	0.088	0.337 **	−0.228 **					
Barriers Self-Efficacy	0.019	0.074	−0.026	−0.220 **	0.179 **				
Positive Outcome Expectations	0.012	0.061	−0.012	−0.083	0.106	0.250 **			
Negative Outcome Expectations	0.073	−0.010	−0.099	0.373 **	−0.217 **	−0.122 *	−0.126 *		
Total Social Support	0.014	0.099	−0.043	−0.251 **	0.091	0.074	0.178 **	−0.035	

Note: ** Correlation is significant at the 0.01 level (2-tailed). * Correlation is significant at the 0.05 level (2-tailed). Abbreviations: BMI, body mass index; MVPA, moderate-to-vigorous PA.

**Table 5:** Multiple variable linear regression analyses using BMI as the dependent variable.

Variable	B	β	*p*-Value
Constant	36.717		<0.001
Exercise Barriers Interference	0.013	0.022	0.677
Walking Self-Efficacy	−0.052	−0.182	0.001
Negative Outcome Expectations	−0.103	−0.038	0.474
Cardiorespiratory Fitness	−0.375	−0.259	<0.001
Comorbidity Score	0.745	0.184	<0.001
WOMAC	0.042	0.086	0.135
Income	0.247	0.016	0.756
Race	2.947	0.141	0.008
Education	−0.167	−0.009	0.859

Note: B represents the unstandardized regression coefficient; β represents the standardized regression coefficient. Abbreviations: WOMAC, Western Ontario and McMaster Universities osteoarthritis index score.

## Data Availability

Not applicable.
